# Kaposi’s Varicelliform Eruption After Treatment With Ixekizumab in a Patient With Pityriasis Rubra Pilaris

**DOI:** 10.7759/cureus.38395

**Published:** 2023-05-01

**Authors:** Jana S Guenther, Iris Ahronowitz, Scott Worswick

**Affiliations:** 1 Department of Dermatology, University of Southern California Keck School of Medicine, Los Angeles, USA

**Keywords:** il-17 inhibitor therapy, biologic treatment, ixekizumab, herpes simplex virus, kaposi’s varicelliform eruption, pityriasis rubra pilaris

## Abstract

Pityriasis rubra pilaris (PRP) is a rare condition characterized by red-orange plaques with islands of sparing with follicular and palmoplantar hyperkeratosis. The disease can be difficult to treat and often requires patients to trial multiple therapeutic options. In recent years, targeted biologic therapies have increasingly been trialed due to their relative efficacy and favorable safety profile. Ixekizumab, an interleukin-17 inhibitor, is one such therapy that has demonstrated efficacy in PRP with few reported adverse events. We present a PRP patient who developed Kaposi’s varicelliform eruption followed by a pseudomonal superinfection three months after initiation of ixekizumab.

## Introduction

Pityriasis rubra pilaris (PRP) is a rare inflammatory papulosquamous disorder whose pathogenesis is incompletely understood. It is divided into six subtypes with common clinical features including red-orange plaques with islands of sparing with follicular and palmoplantar hyperkeratosis [1,2]. In some cases, it can progress to general erythroderma. Because it is a heterogenous disease without a universal approach to treatment, many patients trial multiple therapeutic options before achieving disease remission [3]. Targeted biologic therapies are increasingly being used in the treatment of recalcitrant PRP given multiple case reports and series describing the efficacy and relative safety of these drugs [3].

Ixekizumab, a monoclonal antibody targeting interleukin (IL)-17, has been approved for the treatment of psoriasis, psoriatic arthritis, and ankylosing spondylitis and has recently been found to be effective in some cases of PRP, including in one clinical trial [4,5]. It has a favorable safety profile. However, as with other IL-17 inhibitors, it does carry a heightened risk for herpes simplex virus (HSV) infections [4,5]. This report describes a patient with PRP that developed Kaposi’s varicelliform eruption (KVE), a disseminated HSV infection in compromised skin, after initiating treatment with ixekizumab.

## Case presentation

A 60-year-old man with a two-year history of recalcitrant erythrodermic biopsy-proven PRP presented to the outpatient dermatology clinic with a tender new flexural rash. At the time of presentation, he was 12 weeks into a trial of ixekizumab 80 mg subcutaneous injection every two weeks for treatment of his PRP and had transitioned to monthly dosing. His most recent dose was administered the week of the presentation. He was also using topical triamcinolone 0.1% cream for his body, desonide 0.05% cream for his face, gabapentin 300-1500 mg as needed for pain, and naltrexone 4.5 mg daily as well as a three-day course of aprepitant 120 mg/80 mg/80 mg monthly for pruritus. His PRP had previously been treated with phototherapy, methotrexate, apremilast, and acitretin without significant improvement. The most beneficial treatment to that point had been topical triamcinolone cream, which he used throughout his disease course.

Initiation of ixekizumab for his PRP had resulted in mild improvement of dorsal hand erythema and palmar keratoderma within four weeks, but improvement plateaued without further improvement thereafter. Eight weeks after starting treatment, the patient developed tenderness in his bilateral axillae and inguinal region. He then contracted COVID-19 pneumonia, for which he was treated with a course of doxycycline by his primary care provider.

**Figure 1:**
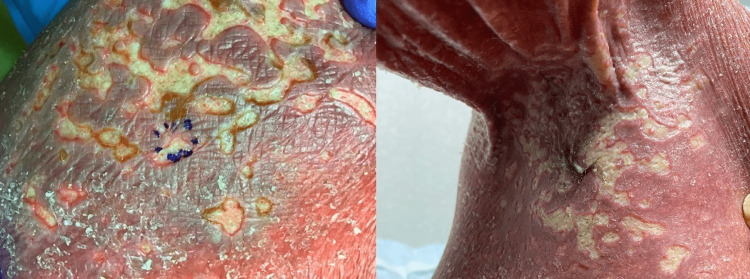
Confluent punched-out erosions of the inguinal region (left) and axilla (right)

Upon presentation to the clinic, the patient reported the development of extraordinarily tender pustules and ulcers in the axillae and inguinal area over the past week without systemic symptoms. Physical examination revealed numerous confluent punched-out erosions of the bilateral axillae, upper medial thighs, inguinal region, scrotum, left outer hip, and bilateral shins (Figure 1).

**Figure 2:**
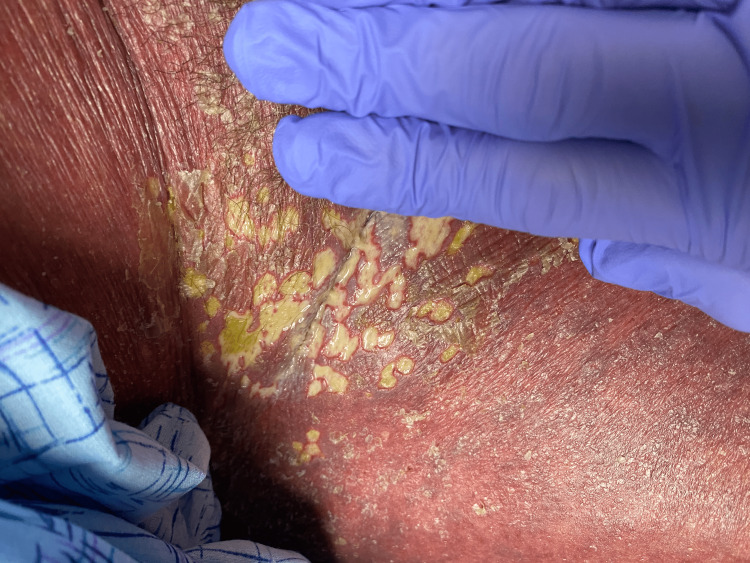
Green-tinged exudate coating erosions of the inguinal region

No pustules or vesicles were seen. A skin biopsy only revealed an ulcer without a readily identifiable cause. However, a viral culture was positive for HSV-1. Therefore, the patient was prescribed 1 gram of oral valacyclovir twice daily for 14 days. Close follow-up on the fourth day of treatment revealed that erosions had stopped progressing and some had started healing. However, the patient continued to complain of severe pain, for which his standing gabapentin dose was increased. Additionally, thick green-tinged exudate was noted to be coating the inguinal ulcers (Figure 2), and bacterial culture confirmed suspected *Pseudomonas aeruginosa (P. aeruginosa*) infection.

**Figure 3:**
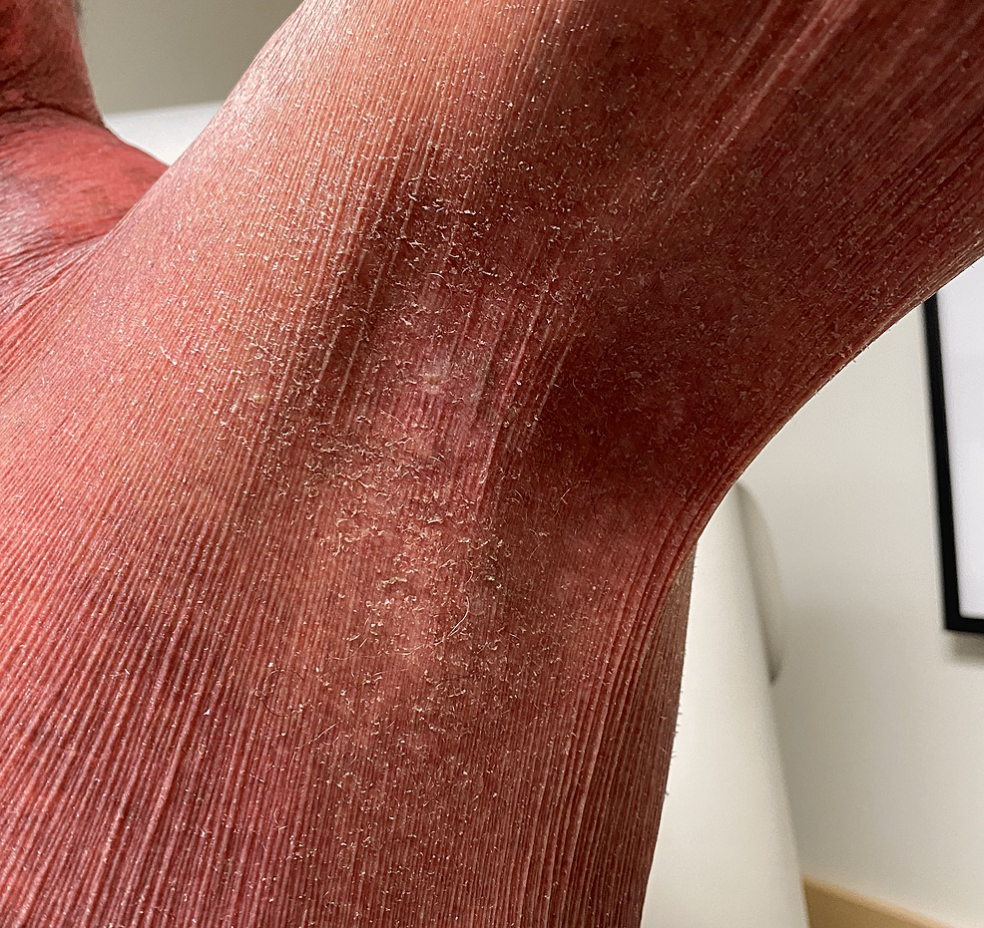
Healed erosions after treatment with valacyclovir

The patient required two courses of ciprofloxacin 500 mg orally twice per day for 10 days each to resolve the *P. aeruginosa* infection. HSV-1 infection required a second 14-day course of valacyclovir 1 gram twice daily to achieve resolution (Figure 3). The patient was then transitioned from ixekizumab to ustekinumab given the lack of improvement in his PRP and the development of KVE.

## Discussion

KVE is a disseminated viral skin infection, most often due to HSV, superimposed upon a preexisting skin condition [6]. It presents as an eruption of closely grouped vesicles and pustules that commonly evolve into punched-out erosions [6]. When the infection occurs over pre-existing atopic dermatitis, it is often referred to as “eczema herpeticum" [6].

KVE with underlying PRP is not commonly described, with only three previously reported cases in the literature [7-9]. Compared to these previous cases, in which patients developed KVE within weeks to months after PRP diagnosis, our patient had a much longer PRP disease course prior to infection. Despite two years of treatment with immune-suppressing agents, no HSV infection had previously developed. In two of three previously reported cases, patients had received recent treatment with UVB phototherapy, systemic corticosteroids, or acitretin, none of which our patient was receiving at the time of infection [7,8]. Instead, he was using topical corticosteroids, which had been a mainstay of his treatment over the course of the disease, and newly commenced ixekizumab.

Notably, superinfection of HSV with *P. aeruginosa* is also rarely reported. Two reports have described its occurrence in patients with underlying atopic dermatitis [10,11], and one described superinfection of recurrent HSV-2 [12]. Its development in our patient is likely attributed to the combination of IL-17A blockage and defective skin barrier.

Several case studies have described the successful use of ixekizumab for the treatment of PRP [13-16]. The only clinical trial of its use in PRP, a single-arm study of 12 patients, found that ixekizumab was associated with reduced clinical signs and symptoms of PRP in a subset of patients, including those with refractory disease [4]. Of the 11 patients that completed treatment, seven achieved a psoriasis area and severity index of 75 or greater. Unfortunately, our patient had only minimal improvement after 12 weeks of treatment.

Ixekizumab has a generally favorable safety profile. The most common adverse reactions to treatment are injection site reactions, upper respiratory tract infections, nausea, and tinea infections [5]. No adverse events have been identified in case reports of its use in PRP. The most frequently reported adverse event in the previously-described clinical trial was an upper respiratory tract infection in four patients [4]. No cases of herpes simplex or herpes zoster were reported. In a clinical trial of 206 patients receiving ixekizumab for moderate-to-severe psoriasis over five years, seven patients reported herpes simplex infection and eight reported herpes zoster infection, all of which were mild or moderate except for one severe case of herpes zoster [5].

The role of IL-17 in viral infection is still under investigation. A study of IL-17A knockout mice found IL-17A to play a role in enhancing antiviral T helper type 1 response to HSV-2 reinfection in the female genital tract [17]. The impaired immune response in these mice resulted in increased disease severity and mortality compared with wild-type mice [17]. In another study, treatment of human vaginal epithelial cells with IL-17A resulted in diminished HSV-2 replication [18]. Such results suggest the possible involvement of IL-17 in the immune response against HSV.

It is of note that our patient was infected with COVID-19 two weeks prior to the development of KVE. An association has been made between HSV-1 reactivation and COVID-19 infection, though the nature of the association has not been elucidated [19]. We speculate that COVID-19 infection in combination with ixekizumab may have lowered the threshold for the development of disseminated HSV reactivation in this patient, though further studies are required to evaluate this observation.

## Conclusions

KVE is not commonly reported in patients with PRP, but it is an important diagnosis to consider as prompt antiviral therapy is crucial for the treatment of the condition. In the case of our patient, ixekizumab may have contributed to the development of KVE. Although data on the use of ixekizumab for PRP have thus far been promising, further studies and reports of its use in PRP are necessary to fully understand its efficacy and potential risks.
